# How community engagement strategies shape participation in mass drug administration programmes for lymphatic filariasis: The case of Luangwa District, Zambia

**DOI:** 10.1371/journal.pntd.0007861

**Published:** 2019-11-27

**Authors:** Adam Silumbwe, Hikabasa Halwindi, Joseph Mumba Zulu

**Affiliations:** 1 Department of Health Policy and Management, School of Public Health, University of Zambia, Lusaka, Zambia; 2 Department of Community and Family Medicine, School of Public Health, University of Zambia, Lusaka, Zambia; 3 Department of Health Promotion, School of Public Health, University of Zambia, Lusaka, Zambia; Erasmus MC, NETHERLANDS

## Abstract

**Background:**

The success of the global strategy to eliminate lymphatic filariasis (LF) through mass drug administration (MDA) campaigns is dependent on meeting high coverage levels over long periods of time. Community engagement plays a critical role in driving coverage and involvement of local communities in MDA for LF. This study explored how community engagement approaches used in MDA for LF shape participation in the programme, with a view of proposing effective engagement strategies.

**Methods:**

The study was conducted in Luangwa, a rural District of Lusaka province, Zambia. An exploratory qualitative case study approach was employed. A total of nine focus group discussions, six in-depth and seven key informant interviews were conducted with various participants that included; community members, traditional leaders and programme managers, respectively. Data were analysed using a thematic approach, aided by NVivo 10 software.

**Results:**

Three core thematic areas emerged from the data as priority focus areas for programme planners and implementers in designing effective community engagement strategies that facilitate participation. Firstly, employing of partnership approaches through adequate and timely engagement of traditional, government and non-governmental organisation structures. Secondly, use of appropriate and innovative health education initiatives to disseminate information about the programme. Thirdly, addressing context specific programme implementation barriers affecting community engagement in MDA for LF.

**Conclusion:**

Facilitating participation in MDA for LF will require designing and implementing effective community engagement strategies that take into account local context, but also seek to explore all avenues of maximizing participation for improved coverage levels. MDA for LF implementation teams should systematically consider the identified factors and seek to incorporate them in their implementation plans.

## Author summary

The lymphatic filariasis (LF) parasite is highly prevalent in many parts of Zambia, with almost 10 million people at risk of infection. Country wide mapping between 2003 and 2011 showed a high prevalence ranging from 1–54% of the circulating filarial antigen, dictating the implementation of mass drug administration (MDA). MDA for LF was first piloted in Kalabo District, Western Province in 2015 and was later scaled up to other provinces in 2016. MDA for LF programmes have reported lots of challenges in attaining required coverage levels in many settings, particularly during the early rounds of implementation. Community engagement processes during MDA for LF programme implementation have a key role to play in driving coverage and participation. However, engagement processes and their influence on participation in MDA for LF programs are rarely documented in most instances. This study was conducted in 2017 with a key focus on the engagement process employed in the first (2016) and second rounds (2017) of MDA for LF and how they influenced participation in the programme. We also sought to identify some key constraints to engagement of local communities in the study site, Luangwa district, Lusaka Province.

## Introduction

Lymphatic filariasis (LF) has been recognised as a global public health problem affecting close to a billion people in most low resource settings[1]. Sub-Saharan Africa (SSA) reports a substantial proportion of this burden, thus resulting in huge economic losses and disability due to the disease[2–4]. To eliminate LF, the World Health Organization (WHO) recommends implementation of mass drug administration (MDA) in endemic countries, for a period of at least five years, with consistent high drug coverage levels above 65% of the population at risk [5].

In Zambia, about 10 million people are at risk of infection with LF [6]. The mapping of the circulating filarial antigen (CFA) estimated a prevalence ranging from 1% to 54% [7]. These high prevalence levels have dictated implementation of MDA as recommended by the WHO, for the period 2015–2019. The period falls a year short of the WHO global target to have LF eliminated as a public health problem by the year 2020[8]. This entails that Zambia has a relatively constrained timeframe to implement the MDA for LF programme, which requires an accelerated implementation plan that consistently meets and sustains the WHO annual drug coverage threshold [9]. It also requires the putting in place of effective engagement strategies that encourage high levels of participation and compliance[10].

The MDA for LF programme in Zambia is under the Neglected Tropical Diseases Unit, Department of Public Health, at the Ministry of Health. Implementation of the programme is done through the District health office and local health centres for a five days period every year. Community members are administered with an annual dosage of diethylcarbamazine citrate (6 mg/kg) and albendazole (400 mg) by the community drug distributors (CDDs). These are trained community health workers who deliver drugs to the households under supervision of the health facility staff. The CCDs are also responsible for social mobilisation through provision of information, education and communication materials to community members. Between 2016–2017, the MDA for LF programme reported national drug coverage levels of above 75%[11].

Though participation in the MDA for LF programme is driven by various factors, no Zambian study has specifically documented the role of community engagement. Successful undertaking of MDA for LF requires that communities are actively engaged [12, 13], as individuals may be wary of participating owing to various community level factors [14–16]. Community engagement processes that promote participation are essential to achieving sustainable and successful implementation of MDA for LF[17]. They provide an opportunity for improved awareness creation, community empowerment and facilitate programme ownership by the communities[18]. Consequently, it is important that much attention be dedicated to the processes by which communities are engaged in MDA for LF campaigns as it has huge implications on their ability to participate [19, 20].

Despite the wide documentation of the pertinent role engagement processes play in shaping participation in MDA for LF campaigns, literature from several other sub-Saharan African countries has shown that there is limited emphasis on understanding community engagement processes and their linkage to participation. This was a key highlight of the systematic review conducted by the authors on factors that shape implementation of MDA for LF in sub-Saharan Africa[21]. Other studies have also found that implementation challenges such as limited involvement of local communities and unclear strategies for effective advocacy affect participation in MDA for LF [9, 22].

In this regard, there indeed remains much scope to explore how MDA for LF programmes are engaging communities, where implementation research can help address barriers and propose new strategies preferably as part of a long-term and sustainable engagement strategy [23]. This study therefore sought to document the community engagement processes and how they shaped participation in the first and second round of MDA for LF in Luangwa District of Zambia (2016–2017), with a view of proposing an effective community engagement strategy.

## Methodology

### Study design

An implementation research approach that included involving the programme people responsible for Neglected Tropical Diseases (NTD) at the Ministry of Health at all stages of the study was employed. This was a qualitative case study design that sought to understand the engagement strategies used in MDA for LF implementation and how they interact with contextual factors to influence participation in the programme in Luangwa district of Zambia. This design helped to critically look at the case of Luangwa district and identify key insights into community engagement strategies for other implementers in similar settings.

### Study setting

We conducted this study in Luangwa District of Lusaka Province, Rural South-East Zambia[24]. The district is located in a Rift Valley, at the confluence of the Zambezi and Luangwa rivers at altitudes below 600 m above sea level. The main source of livelihood in the district is fishing on the rivers, production of reed mats and subsistence farming. The study site was identified based on findings from an LF mapping survey carried out in the area in 2011, when it recorded a CFA prevalence of 33.3%, the highest in the province. Data was collected based on the second round of MDA for LF in 2017. Whilst the MDA for LF programme is stated to be between 2015–2019, the first year was considered a pilot, and scaling to other parts of the country was done from 2016 onwards.

### Study population

The study population consisted of people that participated in at least one MDA for LF campaign between 2016 to 2017. Study participants had to be residents of the MDA for LF target area and were at least above the age of 18 years. The healthcare providers and other key community stakeholders involved in the programme were also sampled.

### Sampling and recruitment of participants

Purposive sampling was used to select study participants. Though purposive, the recruitment ensured a participatory approach by engaging a local district health coordinator who was recommended by the District Health Office. The coordinator worked with the nursing sister in-charges at the health facilities, together with some community health representative groupings in recruiting participants. Luangwa District has three principle health facilities in three zones that were the main distribution points for MDA: namely Luangwa BOMA clinic (urban), Chitope (rural) and Mpuka (rural) clinics. We sampled all three facilities as our study sites.

### Data collection

Six in-depth interviews (IDIs) were conducted. The selection of IDI participants was guided by the programme implementers and community members, based on the participants roles in community engagement during implementation of MDA for LF (Table 1). Seven key informant interviews (KIIs) were conducted with facility in-charges and programme coordinators selected from each of the three study sites, and the overall district coordinator (Table 2). Nine focus group discussions (FGDs) were held with the community members, three per study site comprising 7–10 members per group. We had two groups of community members categorised according to whether they were adolescents or adults and another one for the community drug distributors (CDDs). All CDDs working at the study sites that participated in MDA for LF were selected. In total, nine FGDs, with 69 participants were conducted across the three study sites (Table 3). Theoretical saturation of information from all the categories of participants further guided both the number of FGDs as well as the interviews that where conducted.

**Table 1:** In-depth interviews.

Participants	Number of interviews
Religious leadership	1
Traditional leadership	1
Community development	1
Child fund	1
School health and nutrition coordinator (SHIN)	1
Zambia information services (ZANIS)	1
**Total number of interviews**	**6**

**Table 2:** Key informant interviews.

Participants	Number of interviews
Facility in-charges	3
Facility programme coordinators	3
District health office	1
**Total number of KIIs**	**7**

**Table 3:** Focus group discussions.

Sites	Focus group discussions	Number of participants
Luangwa BOMA clinic (Urban)	Adolescents (18–19 years)	7
	Adults (>19 years)	10
	Community distributors	8
Mpuka rural health centre	Adolescents (18–19 years)	8
	Adults (>19 years)	8
	Community distributors	7
Chitope rural health centre	Adolescents (18–19 years)	7
	Adults (>19 years)	7
	Community distributors	7
**Total FGD participants**		**69**

### Data management and analysis

Thematic approach, a method for identifying themes within data was used for data analysis [25]. NVivo 10 software by QSR international was used to manage and explore the data. The software facilitated identification of themes by running queries that allowed identification of patterns within the coded qualitative data. The data analysis process started with a collection of information gathered through field notes. Interviews and FGDs were audio recorded in local language Nyanja, with participant permission, and transcribed and translated verbatim into English. A code-list iteratively developed from the field notes and more detailed reading of the transcribed data is presented below (Table 4). The code-list provided the basis for structured data analysis and clear visualisation of the relationship amongst the themes.

**Table 4:** Qualitative data analysis code-list.

Broader categories	Analytical node 1	Analytical node 2
Partnership approaches		
	Network of Community health workers	
	Traditional and religious leadership	
	District development committee	
**Innovative health education initiatives**		
	Community meetings and public address system	
	Radio announcements and mobile phones	
	Information education and communication materials	
	Community response to MDA for LF health education	
		Knowledge about MDA for LF
		Community decision to take the drugs
		Practices of LF patients after health education
**Context specific implementation barriers affecting engagement**		
	Implementation period of MDA for LF	
	Mobile populations in Luangwa district	
	Fear of drug side effects	
	Morbidity and disability prevention services	
	Incentives for community drug distributors	

### Ethical considerations

This study received ethical approval from the University of Zambia Biomedical Research Ethics Committee (REF. No. 013-07-26), and all prerequisite authorizations were obtained from the Ministry of Health. All participants (>18 years) provided written, informed consent to participate in the study. In the event that participants who were not literate, a witness was required to be present during the consenting process and sign consent on their behalf. The participants gave separate consent to being audio recorded.

## Results

The qualitative results are presented with the relevant verbatim quotes according to the three thematic areas that emerged from the data. These three thematic areas identify focus areas for enhancing community engagement in MDA for LF. Whilst data were collected across various participant categories, no major differences in the discussions were noted. In instances where views are specific to a particular group, these are noted within the manuscript.

### Partnership approaches to implementation of mass drug administration for lymphatic filariasis

#### Local network of community health workers (CHWs)

Both the community members and healthcare providers stated that community health structures were the starting point in ensuring participation in MDA for LF. These local networks of CHWs were responsible for conducting health education and drug distribution during MDA for LF. Community members however, emphasised the importance of CHWs having to hail from the same community where they were assigned to conduct health education and drug distribution, so that people could trust and refer to them after the MDA campaign. Furthermore, community members stressed the need for drug distributors to be knowledgeable about LF, so that they can confidently respond to any queries about the drugs.

“…We have people that volunteer to be part of a health facility, to help in extending services to the community. When it comes to MDA for LF, we use them to distribute the drugs and conduct health education. The main reason is that they are known to the community as representing the health facility, so they can be trusted…” [KIIB2_ Healthcare provider]

#### Traditional and religious leadership structures

Traditional and religious leadership structures were reported to be important in facilitating participation in MDA for LF. Churches for example, were said to command huge followings in most of the communities, and were therefore engaged in the provision of health education. The healthcare providers recounted that they sent letters to the local churches explaining the essence of MDA for LF and requested that they encourage their members to participate. Some community members further conveyed that they had gotten information about MDA for LF from the churches, and were encouraged to take the drugs.

“…We usually talk to the churches. We realised that traditional leaders have a bigger voice, and maybe even be bigger than us. As you are aware, people will usually believe a pastor or an elder than a health worker. So, we decided that one of the ways we are going to engage the community is to use the religious leaders…” [KII1_ Healthcare provider]

#### District development committee

Other government departments had a pivotal role in enhancing participation in MDA for LF. The Luangwa district development committee provided a platform for linkages between the district health office and other government departments. It allowed for the coordination of development efforts from all key stakeholders in the district including the department of health, local government, education, agriculture and fisheries and community development. With regards to MDA for LF implementation, the committee was fundamental in fostering a multi-sectoral approach, local support and sharing of resources from the onset.

“…Before administration of the drug, stakeholder meetings through the district development committee were held with traditional and civic leader, district heads of departments and the community to explain what MDA for LF entails…” [IDI1_Key stakeholder]

Through the committee, key stakeholders such as civic leaders were also used to promote stakeholder buy-in and generation of political-will for the programme. These influential people played an important role in motivating the community members to participate in MDA for LF. In the local schools for example, the district education board was engaged to facilitate for the process of allowing teachers to participate in conducting health education and drug distribution in their institutions. This was critical in facilitating participation of students in the schools.

“…We engage those influential people. For example, the civic leaders such as the counsellors and council chairperson. Then we have the senior people in the government who are the DC, council secretary and the DEBs. So, we usually have what we call the stakeholders’ meetings with them where we present what lymphatic filariasis is, the background and then the benefits to the community. So, from the onset we build the foundation where they understand why it’s necessary…” [KII1_Healthcare provider]

### Appropriate and innovative health education initiatives

#### Door-to-door, drama and IEC materials

Awareness creation strategies included the use of the door-to-door approach to conduct health education. This is where the CDDs moved from house-to-house educating people about MDA for LF. Drama was also used in instances when they were sufficient financial resources to hire performers. Community members were made to gather in a particular location, where key LF diseases aspects were dramatized. Furthermore, Information education materials such as posters and leaflets were used for health education. These where stacked in selected places such as the health facility, and some were given to the CDDs as references when conducting health education. However, the CDDs stated that most of these health promotion materials did not cater for every household they visited. They also indicated the need to have some IEC materials translated to local languages to enable community members better understand the importance of MDA for LF.

“…Through the drama groups, people were informed. When they performed, they educated the people because the drama was based on the disease. Drama tends to bring a lot of people together, and after that they provide them with health education and people do learn…” [FGDB6_ CDDs]

“…We only knew about elephantiasis after we were shown pictures, which showed swelling of one breast, legs and deformation of body parts. I heard that a specific mosquito causes elephantiasis and one has to take the drugs before they acquire the disease and that is how we took the drugs…” [FGDC4_ Adolescent]

#### Community meetings and public-address system

Community meetings were used to create awareness. These were called by community leaders such as the headmen, neighbourhood health committees and primary health centres. In these meetings, community members were informed about MDA for LF and encouraged to participate as well as inform others about the programme. The public-address system was also used in certain areas to make announcements on the need for communities participate in the programme. However, the use of the public-address system depended on the availability of funds, whilst community meetings were stated not to be the best awareness creation platforms because some community members absconded.

“…They are community meetings called by influential leaders like the headmen where people are informed about MDA for LF. There is also a public-address system used to sensitize people. When they are sufficient funds, they do also drama. They also go door–to-door educating the households…” [KII3_Healthcare provider]

#### Mobile phones and radio announcements

Community members suggested the need to use innovative awareness creation approaches to reach as many people as possible. They proposed the use of mobile phones to send text messages about MDA for LF in areas with a local network. Additionally, they suggested that use of neighbouring countries’ community radio stations would help inform people who conducted business outside the district. Once such people were made aware of the program, they could easily plan their activities and avoid missing the drug distribution days. It was further suggested that engaging LF experts to discuss with community members on radio programs would also help create understanding and combat any negative beliefs regarding the drugs.

“…Another way for those who have no radio or TVs, we can use the mobile phones like the way we receive health tips. They can arrange with the network providers and send the text messages about MDA for LF to everyone…” [FGDM4_ Adolescent]

“…One of the ways is to bring us experts in this field to talk to the community members through the headmen and volunteers, who can now do the teaching their communities. Since they are from the same communities, it’s easy even for people to follow them at their houses and consult…” [KII7_ Healthcare provider]

### Community response to MDA for LF health education

#### Community knowledge about MDA for LF

Most of the community members had a good understanding of LF. They defined LF as the abnormal swelling of body parts such as the limbs and genital organs. They were able to explain the cause of the disease together with its symptoms, though they could not distinguish the different chronic manifestations. Similarly, community members were knowledgeable of the essence of MDA for LF and its benefits. However, they stated that it was difficult to ascertain how helpful the drugs were given that those with chronic manifestations of LF could not be cured even after taking the drugs for a long time.

“…Elephantiasis is a disease whereby one part of the body enlarges more than the other, take for example, the hand; one side may become extremely larger than the other. Even the breast, you find breast may be larger than the other…” [FGDC3_Adolescents]

“…To say the truth, it’s very difficult to know whether these drugs are helpful because we have old cases of people with swollen body parts and they have not necessarily reduced even after taking the drugs…” [FGDB7_ Adult]

#### Community decision to take the drugs

Pictures of LF patients were effective in convincing people to take the drugs. Community members narrated that they were motivated to take the drugs after seeing pictures of LF patients during the health education campaigns. They indicated that they were scared of acquiring the disease so they had to take the drugs. Others reported that they took the drugs because they were scared the disease would cause a lot of suffering in their family if any of their family members acquired it.

“…. When the CDDs were distributing, they showed us pictures and brochures of LF patients with swollen breast and hydroceles, which was scary and made us to ensure that all our family members took the drugs…” [FGDC5_ Adult]

#### Practices of LF patients

The community members explained that before the implementation of MDA for LF, most of those with visible chronic manifestation of LF did not seek medical help. This was because they believed that they were bewitched, hence they resorted to visiting witch-doctors hoping to be cured. Others stated that some LF patients used to think it was a family disease passed on from one generation to another, so they ended up staying at home without seeking medical care. However, after the introduction of MDA for LF, community members narrated that most LF patients had gotten more knowledge about the disease and had begun seeking medical assistance at the local health facility.

“…In the olden days LF patients would sit at home without medical care. They visited witchdoctors who eventually tattooed the swollen body parts with the intention to reduce the swellings. For a swollen leg, they tattooed the entire back and similarly for the hydrocele. But nowadays people are going to the hospital…” [FGDC4_CDDs]

### Addressing context-specific programme implementation barriers

#### Short timeframe for implementation of MDA for LF

The period dedicated to implementing MDA for LF was reported to be short by both the healthcare providers and CDDs. They stated that five days of implementing was inadequate to ensure the MDA was done effectively. It was suggested that extending to 2 weeks or 10 days would suffice to cover every area in the district. It would also allow for allocation of adequate time to implementation and social mobilisation activities like conduction of health education campaigns before the actual drug distribution. This would also allow for capturing of community members that may otherwise have missed the drug distribution days.

“…Even the drug distribution time will require a week and some days, because this enables to capture even those that did not get the MDA for LF message. The days should be extended…” Community member [FGDC7_ CDDs]

“…I would also recommend that the timeframe be extended from a one week program to two weeks program or maybe to 10 days, at least it can help to cover quiet a large population…” [KII6_Healthcare provider]

The healthcare providers further narrated that usually, national orders to implement MDA for LF came at very short notice. This was because the programme had no fixed date during the year which occasionally resulted in preparation activities coinciding with other district health programmes. Because of this, the CDDs recounted that they had to overwork themselves in order to meet the set targets, while sharing tasks with other programmes. They had to conduct health education simultaneously with the drug distribution.

“…The period for the drug distribution is very little for us to be able to make follow-ups. We have to panic for us to reach the set targets or percentages. As a result, we are overwhelmed by the work. If the period can be increased so that we can reach the set MDA for LF targets very well…” [FGDB6_CDDs]

“…In the time frame that we had it was not enough I can say to adequately give information. You resort to just saying that people from the facility are coming to your place, but not spend much time with community members because you want to cover everyone…” [KII4_Healthcare provider]

### Mobile populations in Luangwa district

A good number of community members were reported to be fishermen and women, who spent most of their time camping and doing business in the neighbouring countries of Zimbabwe and Mozambique. As a result, they would miss the actual drug distribution days; hence, they would not participate in MDA for LF. This was because they spent long periods of time away from their homes, and they would not be aware of the programme, only to return when it had already been implemented.

“…You find that for those who might have gone to Mozambique like in this area we are bordering Mozambique and Zimbabwe and most of our people here that’s where they do their businesses. They go and buy fish, maybe some other things, meaning that those who are absents during that week, like those selling fish, maybe missed during the five-day period given for drug distribution…” [KII6_Healthcare provider]

### Fear of drugs side effects

Refusal by some community members to take the drugs was another challenge to MDA for LF. Various reasons were reported, but mostly it was due to fear of side effects, personal beliefs and general lack of information about LF treatment in some instances. Some community members were afraid that the drugs would make them drowsy, hungry and vomit. These side effects were commonly reported by all the categories of participants.

“…The other challenge was that some people refused to take the drugs. Even if they were to be in far places, we had to make an effort to follow and convince them to take the medication. They said the drugs made them hungry, sleepy, or it made them to vomit. There is need to educate people so that they can get used…” [FGDB4_ CDDs]

### Lack of morbidity management and disability prevention services

Most of the participants recounted the suffering that LF patients had to go through in their daily lives within the communities. They suggested that whilst MDA was meant for prevention, there was a need to have another programme specifically to help identify those with chronic manifestations of LF and link them to healthcare. Furthermore, both the CDDs and healthcare providers reported that during the drug distribution exercise LF patients would question the rationale of taking the drugs given that they would not be cured.

“…LF patients suffer a lot. We all tend to think they are just swollen body parts, but when you hear the patient talk, they say it’s painful. I experienced that from a close friend who told me that it’s painful sometimes…” [FGDC6_ Adult]

“…We are hoping that as we continue doing it we can even do better. Maybe also try to see how we can help even those who are infected, because sometimes we collect figures, but we don’t provide any help to the people who are infected. So, if we can try to provide assistance to those who are infected wit will be better….” [KII1_Healthcare provider]

“…We have people that have these conditions in this area like lymphedema, they ask to say we continue getting this medication, but what are we getting out of it?” [KII3_Healthcare provider]

### Inadequate incentives for the CDDs

Both the HCPs and community members reported that there was a need to provide appropriate incentives to the CDDs for them to work efficiently. The current financial incentives were thought to be inadequate for the large amount of work that they did. In both of the previous two rounds of MDA for LF, the CDDs conveyed that the money was paid months after they had finished the distribution exercise. This, they said, was demotivating, because they had invested a lot of time that they could have used on other things to benefit their families. Furthermore, incentives such as bicycles to help ease transport challenges were suggested. There was also a suggestion to provide bags for the CDDs to be carrying the drugs.

“…These volunteers offer their energy and resources in capturing information needed by the district. We need some incentives that can help to appreciate their hard work. When we promise them an allowance at the end of each MDA, I think it would be better to give them immediately they are done. That helps to encourage them to do the work better next time, but where we delay to give our dues, it demotivates them…”

[IDI4_Key stakeholder]

## Discussion

The study findings suggest that to attain high levels of participation in MDA for LF, there is need to design and implement effective community engagement strategies. The engagement strategies must aim to address three core issues if they are to be effective in facilitating participation, which include partnership approaches to implementation, appropriate and adequate health education, and addressing context specific implementation barriers affecting community engagement in MDA for LF programme.

The first core thematic area, partnership approaches to MDA for LF implementation is crucial to facilitating participation as it provides a basis for sustained political commitment and support for the programme from government heads of departments and NGOs at both national and district levels. Community partnerships further shape participation and implementation by providing a platform to build social capital, respectful relationships, engender trust and sustain community support towards the MDA for LF programme[26]. Though these findings underscore the importance of local partnerships, strategic international collaborations equally contribute to facilitating participation in MDA for LF as reported in other studies [27–29]. Implementers have therefore to pay equal attention to such opportunities.

The second core thematic area, health education, plays an important role in facilitating participation in MDA for LF. It helps to transform the mind-set of the community through empowering them with information about the relevance of MDA. For such a transformation to occur, it is imperative that sufficient time is allocated to health education, IEC materials are translated into local languages and innovative approaches such as mobile phones are employed when creating awareness. These findings are consistent with similar studies conducted in other parts of sub- Saharan Africa [28, 30]. A study from Sierra Leone showed that use of innovative and more “modern” sensitization approaches, enabled the reaching of individuals and institutions that had otherwise been unaware of MDA for LF[31]. Two Nigerian studies further reported that conducting knowledge attitude and practices (KAP) surveys enabled the MDA for LF programme to design target specific, responsive and widely accepted IEC materials [30, 32]

The third core thematic area, addressing context specific implementation barriers affecting both the demand as well as the supply side of the MDA for LF programme. Sustaining high MDA for LF coverage levels will require that implementation teams take cognizance of community fears of drug side effects, time period dedicated to implementation and incentives paid to frontline workers, the CDDs. It is imperative that the CDDs, who are key to MDA for LF programme success are motivated. The intricate nature of their work in MDAs for LF demands for consistent motivation. Several motivating factors have been suggested by Njomo et al., that include provision of transportation, capacitation and training, proper supervision, trust and familiarity with community and recognition[33].

Enhancing participation and the functioning of local health structures in MDA for LF, will require establishment of formal morbidity management programmes that identify LF patients and link them to care. Whilst the MDA for LF programme’s focus is disease prevention, there is a need for programmes that address the plight of people who have already developed the chronic manifestations of this long-term debilitating condition. Studies from Togo and the island of Zanzibar have shown that Lymphedema management programmes help to maintain community support for MDA for LF through addressing the needs of the individuals in the community with the most visible LF manifestations and providing information about the disease to the family members [27, 34].

Implementing public health interventions such as MDA for LF remains a complicated process, due to limited evidence on how to accurately select and tailor implementation strategies to address the local contextual needs [35]. Existing systematic reviews have provided limited guidance regarding the types of strategies that may be effective in particular circumstances. This research addresses this gap in implementation research by highlighting three thematic areas to focus on when selecting, designing, planning and implementing of effective community engagement strategies that maximise community participation in MDA for LF.

These study findings are of great relevance to MDA for LF Implementation teams. It is important that before the design and actual programme implementation, emphasis is placed on developing effective engagement strategies that maximise participation, as this will be crucial in reaching and sustaining the WHO set effective drug coverage of >75% in all 4–6 rounds of MDA for LF, and ultimately LF elimination. MDA for LF implementation teams should systematically consider some of the factors highlighted above and develop strategies to address them before implementation.

## Strengths and limitations

The study collected data from a wide variety of sources, which enabled for cross-case comparisons and triangulation across the different categories of participants, hence increasing the validity of findings. Furthermore, the qualitative team composed of the student and two supervisors with experience in having conducted various forms of qualitative research work including programme evaluations. One limitation of this study is that it was conducted in a single setting, with a fairly small sample of respondents, which limits the extent to which the findings can be transferable to other settings in a similar implementation context. However, the detailed exploration of how engagement processes shape participation in MDA for LF provides important evidence to enhance implementation efforts of such programmes.

## Conclusions

Facilitating participation in MDA for LF will require designing and implementing effective community engagement plans. In order for this to happen, this study identifies three focus areas which are partnership approaches to MDA for LF implementation, appropriate and adequate health education and addressing context specific implementation barriers affecting both the demand as well as the supply side of the MDA for LF programme. MDA for LF implementation teams should systematically consider these identified factors, determine their relevance to local context and develop a plan to address them prior to implementation.

## References

[1] WHO, Global programme to eliminate lymphatic filariasis: progress report, 2017. Weekly Epidemiological Record., 2018 93(44): p. 589–602.

[2] Gyapong J. and Boatin B., Neglected tropical diseases-sub-Saharan Africa. 2016: Springer.

[3] Bockarie M.J. and Rebollo M.P., Reducing the population requiring interventions against lymphatic filariasis in Africa. The Lancet Global Health, 2016 4(3): p. e154–e155. 10.1016/S2214-109X(15)00292-2 26874545

[4] Hotez P.J. and Kamath A., Neglected tropical diseases in sub-Saharan Africa: review of their prevalence, distribution, and disease burden. PLoS neglected tropical diseases, 2009 3(8): p. e412 10.1371/journal.pntd.0000412 19707588 PMC2727001

[5] Ndeffo-Mbah M.L. and Galvani A.P., Global elimination of lymphatic filariasis. The Lancet Infectious Diseases, 2017 17(4): p. 358–359. 10.1016/S1473-3099(16)30544-8 28012944

[6] Mwase E.T., et al, Mapping the geographical distribution of lymphatic filariasis in Zambia. PLoS Negl Trop Dis, 2014 8(2): p. e2714 10.1371/journal.pntd.0002714 24587466 PMC3930513

[7] Shawa S.T., et al, Lymphatic filariasis in Luangwa District, South-East Zambia. Parasit Vectors, 2013 6(1): p. 299 10.1186/1756-3305-6-299 24499525 PMC3853755

[8] Ottesen E.A., Lymphatic Filariasis: Treatment, Control and Elimination, in Advances in Parasitology, David H.M., Editor. 2006, Academic Press p. 395–441. 10.1016/S0065-308X(05)61010-X 16735170

[9] Bockarie M.J., et al, Preventive chemotherapy as a strategy for elimination of neglected tropical parasitic diseases: endgame challenges. Phil. Trans. R. Soc. B, 2013 368(1623): p. 20120144 10.1098/rstb.2012.0144 23798692 PMC3720042

[10] Allotey P., Reidpath D.D., and Pokhrel S., Social sciences research in neglected tropical diseases 1: the ongoing neglect in the neglected tropical diseases. Health Res Policy Syst, 2010 8(1): p. 32. 20961461 10.1186/1478-4505-8-32 PMC2987896

[11] Sokesi T., et al, Lessons learnt from the implementation of mass drug Administration for Schistosomiasis and Soil-Transmitted Helminths in Lusaka Province, Zambia. Medical Journal of Zambia, 2016 43(2): p. 82–87.

[12] Adhikari B., et al, Community engagement and population coverage in mass anti-malarial administrations: a systematic literature review. Malaria journal, 2016 15(1): p. 523 10.1186/s12936-016-1593-y 27806717 PMC5093999

[13] Sahan K., et al, Community engagement and the social context of targeted malaria treatment: a qualitative study in Kayin (Karen) State, Myanmar. Malaria journal, 2017 16(1): p. 75 10.1186/s12936-017-1718-y 28196536 PMC5310060

[14] Ahorlu C.S., et al, Community perspectives on persistent transmission of lymphatic filariasis in three hotspot districts in Ghana after 15 rounds of mass drug administration: a qualitative assessment. BMC public health, 2018 18(1): p. 238 10.1186/s12889-018-5157-7 29433461 PMC5809858

[15] Njomo D.W., et al, The role of personal opinions and experiences in compliance with mass drug administration for lymphatic filariasis elimination in Kenya. PLoS One, 2012 7(11): p. e48395 10.1371/journal.pone.0048395 23185256 PMC3501492

[16] Kisoka W.J., et al, Community Members’ Perceptions of Mass Drug Administration for Control of Lymphatic Filariasis in Rural and Urban Tanzania. J Biosoc Sci, 2016 48(1): p. 94–112. 10.1017/S0021932015000024 25790081 PMC4668335

[17] Gyapong J.O., et al, Elimination of lymphatic filariasis: current perspectives on mass drug administration. Research and reports in tropical medicine, 2018 9: p. 25 10.2147/RRTM.S125204 30050352 PMC6047620

[18] Atkinson J.-A., et al, The architecture and effect of participation: a systematic review of community participation for communicable disease control and elimination. Implications for malaria elimination. Malaria journal, 2011 10(1): p. 225. 21816085 10.1186/1475-2875-10-225 PMC3171376

[19] Kouassi B.L., et al, Perceptions, knowledge, attitudes and practices for the prevention and control of lymphatic filariasis in Conakry, Republic of Guinea. Acta tropica, 2018 179: p. 109–116. 10.1016/j.actatropica.2017.12.002 29224979

[20] Hussain M.A., et al, Mass drug administration for lymphatic filariasis elimination in a coastal state of India: a study on barriers to coverage and compliance. Infectious diseases of poverty, 2014 3(1): p. 31. 25237478 10.1186/2049-9957-3-31 PMC4166397

[21] Silumbwe A., et al, A systematic review of factors that shape implementation of mass drug administration for lymphatic filariasis in sub-Saharan Africa. BMC public health, 2017 17(1): p. 484 10.1186/s12889-017-4414-5 28532397 PMC5441010

[22] Klepac P., et al, Towards the endgame and beyond: complexities and challenges for the elimination of infectious diseases. 2013, The Royal Society. 10.1098/rstb.2012.0137 PMC3720036 23798686

[23] Allotey P., et al, Efficacious, effective, and embedded interventions: implementation research in infectious disease control. BMC Public Health, 2008 8(1): p. 343. 18826655 10.1186/1471-2458-8-343 PMC2567977

[24] M.o.H.-G.o., Z.C.S.O.C.-G.o.a., Zambia Demographic and Health Survey (ZDHS). 2013–2014.

[25] Braun V. and Clarke V., Using thematic analysis in psychology. Qualitative research in psychology, 2006 3(2): p. 77–101.

[26] Liese B., Rosenberg M., and Schratz A., Programmes, partnerships, and governance for elimination and control of neglected tropical diseases. The Lancet. 375(9708): p. 67–76. 10.1016/S0140-6736(09)61749-9 20109865

[27] Sodahlon Y.K., et al, A success story: Togo is moving toward becoming the first sub-Saharan African nation to eliminate lymphatic filariasis through mass drug administration and countrywide morbidity alleviation. PLoS Negl Trop Dis, 2013 7(4): p. e2080 10.1371/journal.pntd.0002080 23593512 PMC3623714

[28] Dembele M., et al, Implementing preventive chemotherapy through an integrated National Neglected Tropical Disease Control Program in Mali. PLoS Negl Trop Dis, 2012 6(3): p. e1574 10.1371/journal.pntd.0001574 22448294 PMC3308933

[29] Hopkins A., Beyond providing drugs: the Mectizan(R) donation stimulates new strategies in service delivery and in strengthening health systems. Curr Pharm Biotechnol, 2012 13(6): p. 1110–9. 10.2174/138920112800399220 22039801

[30] Richards F.O., et al, Epidemiological and entomological evaluations after six years or more of mass drug administration for lymphatic filariasis elimination in Nigeria. PLoS Negl Trop Dis, 2011 5(10): p. e1346 10.1371/journal.pntd.0001346 22022627 PMC3191131

[31] Hodges M.H., et al, High coverage of mass drug administration for lymphatic filariasis in rural and non-rural settings in the Western Area, Sierra Leone. Parasit Vectors, 2010 3: p. 120 10.1186/1756-3305-3-120 21162751 PMC3018440

[32] Hopkins D.R., et al, Lymphatic filariasis elimination and schistosomiasis control in combination with onchocerciasis control in Nigeria. Am J Trop Med Hyg, 2002 67(3): p. 266–72. 10.4269/ajtmh.2002.67.266 12408665

[33] Njomo D.W., et al, Factors associated with the motivation of community drug distributors in the Lymphatic Filariasis Elimination Programme in Kenya: original research. Southern African Journal of Epidemiology and Infection, 2012 27(2): p. 66–70.

[34] Malecela M.N., et al, Eliminating LF: a progress report from Tanzania. J Lymphoedema, 2009 4: p. 10–12.

[35] Powell B.J., et al, Methods to improve the selection and tailoring of implementation strategies. The journal of behavioral health services & research, 2017 44(2): p. 177–194. 26289563 10.1007/s11414-015-9475-6 PMC4761530

